# Willingness and influencing factors of old-age care mode selection among middle-aged and older adults in Henan Province, China

**DOI:** 10.1186/s12877-023-04559-w

**Published:** 2024-01-18

**Authors:** Weicun Ren, Ruibo He, Clifford Silver Tarimo, Lei Sun, Jvxiao Wu, Liang Zhang

**Affiliations:** 1 School of Political Science and Public Administration, Wuhan University, Wuhan, China; 2 Wuhan University Health Governance Research Centre, Wuhan University, Wuhan, China; 3 School of Finance and Public Administration, Hubei University of Economics, 8 Yangqiaohu Avenue, Jiangxia District, Wuhan City, Hubei Province China; 4 Department of Science and Laboratory Technology, Dares Salaam Institute of Technology, Dares Salaam, Tanzania; 5 Department of Health Management, Sanquan College of Xinxiang Medical University, Xinxiang, China; 6 School of Communication and Journalism, Wuhan University, Wuhan, China

**Keywords:** Old-age care mode, Older adult, Choice, Mediation Effect Model, Concentration index

## Abstract

**Background:**

The choice of old-age care methods or places plays an important role in improving the quality of life and well-being of older adults. This study aimed to analyze the choices of various old-age care modes (OCMs) among middle-aged and older adults (MOA) aged 40 years and older in Henan Province, China, and to explore the influence of personal health status, perspectives on old-age (POA) and external support received on their choices.

**Methods:**

This study analyzed the data from the previous survey which included 911 MOA. The mean comparison method was used to analyze the evaluation of MOA prior to selecting OCMs, and the effect of individual characteristics, external support received, and personal health status on the choice of OCM for MOA was assessed by Logistic regression (LR) and Concentration Index. The Mediation Effect Model was used to explore effect manner and scope of MOA’ POA in their choice of OCM.

**Results:**

The overall scores for MOA on the choice of the home-based, community-family, retirement village, nursing homes OCM were 4.06 ± 0.81, 3.70 ± 0.88, 3.72 ± 0.90, 3.49 ± 0.97, respectively. The LR model indicated that education level, number of children, relationship between family members and the relationship with neighbors affected the choice of OCM for MOA (*P* < 0.05). Difference in OCM selection was relatively the largest based on the individual’s POA (Concentration index = -0.0895 ~ -0.0606), and it was shown to play a mediating role in other factors influencing the choice of OCM for MOA (Mediation effect = -0.002 ~ 0.013).

**Conclusions:**

The evaluation of MOA on choosing a non-home OCM was generally, and the number of children and external support received were shown to have a relatively substantial impact on the choice of OCM among MOA, however, their power was affected by MOA’ POA. Policy makers could encourage the MOA’ selection of non-home OCM by improving the relationship among MOA persons while positively transforming their POA.

**Supplementary Information:**

The online version contains supplementary material available at 10.1186/s12877-023-04559-w.

## Background

The “silver wave” of an aging population is an inevitable trend in the world today, and it is spreading from developed countries to developing countries [1]. At present, the average life expectancy in China is close to 77 years, and the proportion of the old population is expected to exceed 25% by 2050 [2, 3]. Henan Province is an agricultural-newly industrialized province with a total population of nearly 100 million. In 2020, older adults over the age of 60 accounted for 13.5% of the population, the proportion of young adults was 63.5%, and the dependency coefficient of older adults reached 21.3% [4]. The changes in the age structure of the population, family size, social mobility of children and other aspects of society, the daily care, spiritual and emotional needs of older adults have encountered more and more obstacles, a scenario which has inevitably led to the transformation of old-age care mode (OCM) [5]. How to choose an appropriate OCM is now a problem that people, families, governments, and society must address [6].

The provision of older adults’ care services mainly comes from three aspects including the family, government and society [7]. In China, the existing alternatives for OCMs mostly consist of home-based, community-family, retirement villages, and nursing facilities. This is due to variations in the health status and economic situation of older persons [8–10]. As the basic unit of society, home-based OCM is said to be irreplaceable. However, it is particularly necessary to provide and choose a variety of socialized OCM as the life expectancy of the population continues to increase and the fertility rate remains relatively low [11]. The British government, for instance, promotes the transition from traditional “hospitalized” OCM to community OCM, as well as the creation of non-governmental old-age care organizations [12], while Japan primarily pursues the socialization of older adult care through the establishment of a nursing insurance system [13]. In the United States, there are three primary forms of OCMs: multiple home-based OCM, community-based OCM, and professional institutional OCM [14].

Diversification of old-age care methods and services has become a powerful measure for the old-age service system to cope with the aging of the population. When presented with various options, the choice of OCM for older persons is influenced by both their internal and external circumstances [15]. Older adults with good health may be more inclined to home-based OCM, where they can often not only obtain comfort and care, but also meet their autonomy or capacity requirements through family activities [16]. A study conducted in Newcastle-upon-Tyne by Collins N. and colleagues revealed that nursing home patients were weak and had complicated physical and mental health demands [17]. Social support and older adults’ perspectives on old-age (POA) have also been proved to be related to their choice of OCM [18]. Cheng CP et al. found that due to the influence of traditional culture, the older adults in China tend to focus on home-based OCM, while their awareness of socialized and commercial OCM is relatively low [19]. In addition, the literature demonstrates that older individuals’ choice of OCM is dependent on their external environment. The study by Roquebert Q et al. found that 76% of nursing home residents and 55% of community residents receive aids from relatives in activities of daily living [20], while a study by Zueras et al. conducted in Spain revealed that economically active and educated middle-aged women were less likely to believe that the family was responsible for the care of older adult relatives [21].

In addition, a large number of studies have shown that the choice of OCM is also related to various factors such as age, marital status, occupation prior to retirement, education level, and the number of children [22]. In China, Li B et al. found that age is positively correlated with the choice of non-home care institutions by older adults. The older individuals who had a partner demonstrated a personal preference for home-based OCM, in contrast to older individuals who did not have a partner [23]. Yang F et al. discovered that the choice of OCM varies for older adults with varying numbers of children [24]. Similarly, it was discovered that having a college degree and appropriate health insurance can enhance the likelihood of older persons selecting institutional OCM [25]. On the other hand, Hunter N et al. emphasize that consumer choice of old-age care services is driven by a combination of factors such as desire for flexibility in service delivery, optimization of mobility, need for human assistance, security and safety, and interaction [26]. Different OCMs reflect the combination of social, environmental, and personal elements, and the choice of an appropriate OCM has a significant impact on the quality of life of older persons [27].

To sum up, there have been many studies on OCM for older adults, but the majority of these studies have focused on the analysis of the current situation, willingness and influencing factors for the choice of OCM. Limited studies have explored the perspectives of middle-aged individuals on the selection of OCM, the comparison of various OCM, and the correlation between OCM selection affecting factors. In this regard, the novelty of this study is that it objectively and statistically evaluated the choice of middle-aged and older adults (MOA) on the existing four major OCMs, explored the influencing factors in the choice of each OCM, and examined the effective manner and scope of influencing factors in the choice of OCM.

In order to help formulate reasonable and effective older adult care service supply strategies and improve the quality of life of older adults, this study used the mean comparison method, Logistic regression (LR) model and Concentration Index method to explore the current situation, structure and related influencing factors of OCM choices for MOA. The Mediation Effect Model was used to explore the manner and scope of the personal POA among MOA in their choice of OCM.

## Methods

### Data sources

The information about MOA was derived from the survey on the willingness of old-age care for the middle-aged and older adults in Henan Province, which was carried out from June to September 2021. The survey adopted a multi-stage sampling design, and adopted a multi-stage stratified random sampling method to select MOA in Henan Province as the study participants. In the first stage, the 18 provincial cities in Henan Province were used as primary sampling units, and they were divided into 4 groups according to their population and economic conditions, whereas one city was selected from each group. The ratio of urban: rural was 1:1, whereas urban and townships were selected respectively in the third stage. In this stage, the sampling population was divided into 6 layers and a sampling survey was conducted according to the ratio of 1:1 between males and females and the three age groups (40 ~ , 55 ~ , and 70 ~ years old). Inclusion criteria included: (1) Age > 40 years old; (2) Agree and have the ability to participate in the survey; (3) Know the four types of OCM: home-based, community-family, retirement village, nursing homes; (4) Henan Province household registration and have lived in the local area for more than 12 months.

The survey was conducted by a group of undergraduate students majoring in health services and management, and all interviewers underwent a specialized training before conducting the interviews. Consisted primarily of question-and-answer formats, and the questionnaires were filled out on-site by the researchers following in-depth face-to-face interviews. The survey respondents were informed of the relevant circumstances of the survey in advance and agreed to participate in the survey. Questionnaires were checked and entered on the same day after they were collected. If the questionnaires were not in line with the facts or the missing items were more than 10%, they will be regarded as unqualified questionnaires. As assessed by the Bioethics Committee of Sanquan College of Xinxiang Medical University, the content and procedures of the study met the ethical requirements of international and national biomedical research, and did not involve human or animal experiments, hence it was exempted from the formal review procedures. The effective response rate was 94.90%, with 960 surveys distributed, and 911 valid questionnaires recovered.

### Evaluation model

The survey utilized a self-developed questionnaire called the “Survey on the willingness of old-age care for middle-aged and older adults in Henan Province”. The questionnaire was created based on existing research in the field [15, 17, 25, 28], and it comprised four sections: basic information about middle-aged and older adults (MOA), health status and external support, preferences for old-age care (POA), and evaluation of options for care management (OCM). The specific questions included in the questionnaire can be found in the Additional file. The reliability of the questionnaire was assessed using Cronbach’s alpha, and its content validity was evaluated using the Content Validity Index (CVI), both of which exceeded 0.70. Among them, the basic information of MOA included age, gender, place of residence, education attainment, marital status, affordability of medical and pension expenses, and the number of children.

#### Outcome variables

The main study’s outcome variables were the respondents’ evaluation of the choice of four types of OCM: home-based, community-family, retirement village, nursing homes. Among them, home-based OCM refers to the older adults living at home and are mainly cared for by their children; community-family OCM refers to the older adults living at home at night, and the community or institutions within the community are responsible for the care during the day [29]; retirement village OCM refers to a type of service institutions that provide the older adults with their own independent houses, including kitchens, but generally only provide emergency care; nursing homes OCM refers to a type of old-aged care institutions that provide care workers and comprehensive daily life services. The evaluation results of each OCM include five grades which included very suitable, suitable, generally, inappropriate, and very inappropriate. In the analysis, they were assigned 1–5 points according to the Likert 5 grade scoring method [30].

#### Independent variables

The independent variables were divided into three parts: (1) Personal health status which included physical health status, self-care ability, illness status, and who is mainly cared for when sick; (2) Individuals’ POA was obtained by the respondents’ evaluation of the importance of 9 indicators (basic diet and daily life, cultural and recreational activities, professional medical care, the concept of “raising children to prevent aging”, service attitude and quality, price and expenditure, national pension policy, family ideas, and other people’s opinion) in their choice of OCM [18]; (3) External support for MOA obtained from the relationship between family members, relationship with neighbors, and main medical insurance they have.

### Concentration index

As one of the measures of equity, the concentration index is often used to indicate the degree of concentration of a health and health service activity among different geographical or level populations [31]. This study applied it to analyze the overall differences in the choice of OCM for MOA with different health status and perspectives on old-age. The commonly used calculation methods for the concentration index include the geometric method and covariance method [32, 33]. Considering that the evaluation of MOA’ choice of OCM was set as continuous data in this study, the geometric method was used. The concentration index (*G*) of the evaluation of OCM selection was:

1 $${\text{G}} = 1{ - }\sum\limits_{i = 0}^{910} {(x_{i + 1} - x_{i} )(y_{i + 1} + y_{i} )}$$

Among them, *x*_*i*_ represents the cumulative percentage of MOA while *y*_*i*_ stands for the cumulative percentage of the evaluation of OCM selection. The value of the concentration index is -1 to 1. The greater the concentration, the closer the absolute value of the concentration index is to 0 [33].

### Mediation Effect Model

The Mediation Effect Model has been widely used in social science research. It can analyze how the influence of independent variables on dependent variables is achieved through mediating variables, and has become an important statistical method to analyze the relationship between multiple variables [34]. This study uses the Mediation Effect Model to examine the relationship between personal POA, health status and external support, and the evaluation of OCM choice in order to test the potential mediating effect of personal POA on the influence of personal health status and external support on OCM choice. The mediation effect model is as follows:

2 $$Y\:=\:cX\:+\:\varepsilon_1$$

3 $$M\:=\:aX\:+\:\varepsilon_2$$

4 $$Y\:=\:c'\;X\:+\:bM\:+\:\varepsilon_3$$

In the model, *Y* represents the evaluation of choosing one type of OCM, *X* represents the health status and external support, and *M* represents the personal POA. The test steps are as follows: first, regress the model (2) to test the significance of the regression coefficient *c* of the evaluation of OCM, external support and pension methods. If c is significant, perform regression on models (3) and (4) in turn to test the significance of the regression coefficient *a* of the mediating variable personal POA and health status and external support, and the regression coefficient *b* of the evaluation of OCM and the mediating variable personal POA. When both *a* and *b* are significant, if *c'* is not significant, it means that personal POA plays *a* complete mediating effect; if *c'* is significant and *c'* < *c*, it means that personal POA plays a partial mediating role. If at least one of *a* and *b* is insignificant, but the Sobel test results were significant, indicating a significant mediating effect [35].

### Statistical analysis

The mean comparison method was used to analyze the evaluation of MOA on choosing different OCMs. Logistic Regression (LR) and Concentration Index were used to assess the impact of individual characteristics, external support received, and personal health status on the choice of OCM for MOA. The Mediation Effect Model was used to explore the manner and scope of the personal POA of MOA in their choice of OCM. *P* < 0.05 was considered to be statistically significant. Data was entered using Epidata 3.0 software and statistical analysis was performed using Excel 2019 and SPSS 20.0 software.

## Results

### Basic characteristics

The basic characteristics of the research subjects were described in Table 1. Among the MOA who participated in the survey, 51.04% were women and 48.96% were men. 33.48% of participants were under 55 years of age, while 32.05% of participants were 70 or older. Half of the respondents live in rural areas, 54.34% were in primary school and below, and more than 20% of MOA were unmarried or divorced. More than 20% of MOA could afford monthly medical and health expenses of 500 yuan or less, while only 23.71% could afford monthly spending of more than 1,500 yuan. In terms of the number of children, those without children were less than 4%. The analysis also found that there were significant differences in the evaluation of the other three OCMs except home-based OCM among MOA with different marital status and number of children (*P* < 0.05). MOA with different educational levels, affordable medical and pension costs had significant differences in their evaluation of home-based OCM (*P* = 0.001, 0.023).

**Table 1:** Characteristics of the study participants (*N* = 911)

Index	Person (%)	Evaluation of old-age care mode selection *(* $\overline{x} \pm s$***)***			
		HOCM^a^	COCM^b^	Retirement village	Nursing homes
**Age (years)**					
40 < Age < = 55	305(33.48)	4.05 ± 0.846	3.77 ± 0.867	3.74 ± 0.926	3.57 ± 0.940
55 < Age < = 70	314(34.47)	4.05 ± 0.769	3.76 ± 0.813	3.78 ± 0.772	3.50 ± 0.812
Age > 70	292(32.05)	4.10 ± 0.817	3.57 ± 0.959	3.65 ± 0.992	3.38 ± 1.047
*F*		0.393	5.079***^d^	1.574	2.749*
**Sex**					
Male	446(48.96)	4.06 ± 0.815	3.71 ± 0.862	3.70 ± 0.889	3.48 ± 0.966
Female	465(51.04)	4.07 ± 0.806	3.69 ± 0.906	3.75 ± 0.91	3.49 ± 0.972
*t*		-0.154	0.199	-0.746	-0.164
**Residence**					
Urban	456(50.05)	4.06 ± 0.868	3.70 ± 0.940	3.77 ± 0.920	3.56 ± 0.943
Rural	455(49.95)	4.07 ± 0.748	3.70 ± 0.825	3.68 ± 0.877	3.41 ± 0.989
*t*		-0.044	-0.026	1.448	2.315**
**Education level**					
Illiteracy	223(24.48)	4.05 ± 0.795	3.53 ± 0.962	3.63 ± 0.93	3.36 ± 1.065
Primary school	272(29.86)	4.11 ± 0.800	3.64 ± 0.829	3.69 ± 0.894	3.42 ± 0.918
Junior high school	202(22.17)	4.00 ± 0.838	3.69 ± 0.827	3.66 ± 0.851	3.45 ± 0.925
High school/Technical school	118(12.95)	4.00 ± 0.773	3.90 ± 0.767	3.90 ± 0.821	3.66 ± 0.869
College degree and above	96(10.54)	4.19 ± 0.85	4.04 ± 0.972	3.98 ± 0.973	3.81 ± 0.998
*F*		1.387	7.657***	4.115***	5.068***
**Marital status**					
Married	706(77.50)	4.11 ± 0.788	3.77 ± 0.852	3.78 ± 0.868	3.54 ± 0.940
Widowed	56(6.15)	3.97 ± 0.788	3.38 ± 0.934	3.52 ± 0.949	3.21 ± 1.037
Unmarried or divorced, et al	149(16.36)	3.71 ± 1.022	3.61 ± 0.966	3.59 ± 1.058	3.48 ± 1.027
*F*		7.494***	12.763***	5.971***	7.140***
**Medical and pension costs (Yuan/month)**^**c**^					
500 and below	192(21.08)	4.17 ± 0.911	3.78 ± 1.011	3.74 ± 0.993	3.48 ± 1.180
501–1000	297(32.60)	4.06 ± 0.766	3.61 ± 0.867	3.64 ± 0.909	3.36 ± 0.912
1001–1500	206(22.61)	3.93 ± 0.739	3.74 ± 0.724	3.71 ± 0.733	3.55 ± 0.858
1501 and above	216(23.71)	4.11 ± 0.826	3.71 ± 0.921	3.83 ± 0.935	3.59 ± 0.920
*F*		3.208**	1.616	1.984	2.891**
**Number of children**					
No	30(3.29)	4.00 ± 1.050	3.83 ± 0.950	3.87 ± 1.224	3.40 ± 1.276
One	143(15.70)	4.03 ± 0.851	3.86 ± 0.836	3.94 ± 0.918	3.70 ± 0.927
Two	402(44.13)	4.05 ± 0.827	3.72 ± 0.874	3.73 ± 0.901	3.53 ± 0.929
Three	208(22.83)	4.08 ± 0.764	3.65 ± 0.843	3.69 ± 0.787	3.48 ± 0.906
Fore and above	128(14.05)	4.15 ± 0.722	3.49 ± 0.980	3.48 ± 0.905	3.13 ± 1.065
*F*		0.495	3.362***	4.746***	6.631***

^a^*HOCM* Home-based old-age care mode

^b^*COCM* Community-family old-age care mode

^c^Medical and pension costs: Affordable medical and pension costs

^d^***, **, * indicate significant at 0.01, 0.05 and 0.1 level, respectively

### Evaluation of the choice of OCM for MOA

#### Personal health status with the choice of OCM

The analysis based on the health condition of MOA revealed that the evaluation ranges of the four types of OCM by a single type of individual were 4.33 ~ 3.56, 4.04 ~ 3.00, 4.05 ~ 3.22, and 3.86 ~ 2.56, respectively. Significant disparities were in the evaluation of the four types of OCM for MOA with varying physical health states, disease conditions, and those who were mainly cared for when they got sick (*P* < 0.05). There was no significant difference in the evaluation of OCM among MOA with different self-care abilities (*P* > 0.05) see Table 2.

**Table 2:** Personal health status with evaluation of old-age care mode selection

Index	Evaluation of old-age care mode selection *(* $\overline{x} \pm s$*)*			
	HOCM^a^	COCM^b^	Retirement village	Nursing homes
**Physical health**				
Very good	4.33 ± 0.871^***d^	4.04 ± 0.991^***^	3.96 ± 1.037^***^	3.81 ± 1.095^***^
Good	3.98 ± 0.737	3.59 ± 0.757	3.67 ± 0.809	3.38 ± 0.835
Generally	3.90 ± 0.780	3.51 ± 0.738	3.58 ± 0.810	3.26 ± 0.872
Poor	3.81 ± 0.634	3.42 ± 0.857	3.46 ± 0.668	3.34 ± 0.901
Very poor	4.00 ± 1.225	3.00 ± 1.225	3.22 ± 1.202	2.56 ± 0.882
**Self-care skills**				
Fully self-care	4.17 ± 0.825^***^	3.83 ± 0.888^***^	3.78 ± 0.966^**^	3.52 ± 1.014
Some rely on others	3.93 ± 0.721	3.50 ± 0.789	3.65 ± 0.751	3.44 ± 0.869
Dependent on others	3.56 ± 0.854	3.28 ± 1.054	3.51 ± 0.798	3.23 ± 0.922
**Disease**				
Not sick	4.14 ± 0.875^**^	3.86 ± 0.905^***^	3.83 ± 0.959^***^	3.60 ± 1.040^***^
One	3.99 ± 0.759	3.60 ± 0.766	3.69 ± 0.765	3.37 ± 0.805
Two	4.02 ± 0.680	3.59 ± 0.817	3.52 ± 0.781	3.44 ± 0.886
Three and more	3.90 ± 0.667	3.07 ± 0.880	3.45 ± 0.963	3.14 ± 0.974
**Caregiver**^**c**^				
Own	4.29 ± 0.819^***^	4.03 ± 0.982^***^	4.05 ± 0.928^***^	3.86 ± 1.054^***^
Spouse	4.08 ± 0.767	3.75 ± 0.813	3.77 ± 0.854	3.51 ± 0.896
Children	4.05 ± 0.750	3.49 ± 0.839	3.52 ± 0.843	3.22 ± 0.950
Relatives or others	3.71 ± 0.999	3.60 ± 0.961	3.64 ± 1.031	3.59 ± 0.951

^a^*HOCM* Home-based old-age care mode

^b^*COCM* Community-family old-age care mode

^c^Caregiver: Who will take care for then when they got sick

^d^***, **, * indicate significant at 0.01, 0.05 and 0.1 level, respectively

#### Choice of OCM with personal POA

According to the respondents’ evaluation of a type of OCM, individuals whose evaluation results were “suitable” and “very suitable” were deemed willing to choose this type of OCM. Table 3 shows the evaluation of the importance of the indicators by the people who are willing to choose a certain type of OCM. When choosing the way of OCM, the importance score of basic diet and daily life was relatively highest (4.51 ± 0.636, 4.62 ± 0.568, 4.54 ± 0.623, 4.55 ± 0.615); the scores of other people’s opinion was relatively the lowest, which were 3.43 ± 1.151, 3.46 ± 1.194, 3.40 ± 1.190, and 3.47 ± 1.240. The MOA who was willing and unwilling to choose retirement village and nursing homes OCM showed no significant difference in their attitudes towards price and expenditure (*P* > 0.05).

**Table 3:** Individual’s perspectives on old-age with the evaluation of old-age care mode selection

Index	Willing to choose this kind of old-age care mode* (* $\overline{x} \pm s$*)*			
	HOCM^a^	COCM^b^	Retirement village	Nursing homes
Basic diet and daily living	4.51 ± 0.636^***^^c,d^	4.62 ± 0.568^***^	4.54 ± 0.623^***^	4.55 ± 0.615^***^
Cultural and recreational activities	4.14 ± 0.884^***^	4.32 ± 0.770^***^	4.19 ± 0.858^***^	4.28 ± 0.856^***^
Professional Medical Care	4.49 ± 0.664^***^	4.54 ± 0.643^***^	4.48 ± 0.680^***^	4.53 ± 0.642^***^
The concept of “raising children to prevent ageing”	4.07 ± 0.921^***^	4.02 ± 1.004^***^	4.02 ± 0.978^***^	4.12 ± 0.956^***^
Service attitude and quality	4.39 ± 0.686^***^	4.46 ± 0.695^***^	4.39 ± 0.697^***^	4.46 ± 0.682^***^
Price and expenditure	4.28 ± 0.821^***^	4.36 ± 0.797^***^	4.23 ± 0.856	4.25 ± 0.901
National pension policy	4.34 ± 0.766^***^	4.43 ± 0.745^***^	4.36 ± 0.798^***^	4.46 ± 0.727^***^
Family ideas	4.28 ± 0.751^***^	4.33 ± 0.750^***^	4.27 ± 0.776^***^	4.31 ± 0.811^***^
Other people’s opinion	3.43 ± 1.151^***^	3.46 ± 1.194^***^	3.40 ± 1.190^**^	3.47 ± 1.240^***^

^a^*HOCM* Home-based old-age care mode

^b^*COCM* Community-family old-age care mode

^c^Indicates the importance of the corresponding perspective index in the choice of old-age care mode that the respondents who are willing to choose a kind of old-age care mode

^d^***, **, * indicate significant at 0.01, 0.05 and 0.1 level, respectively

#### The received external support status with OCM selection

**Fig. 1:**
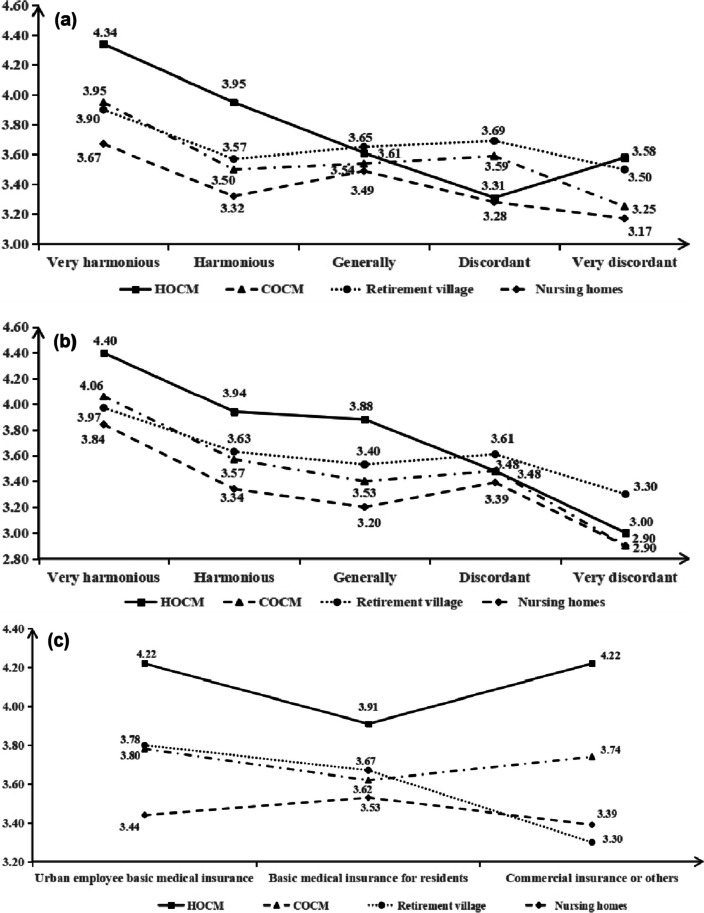
The MOA’ received external support status with their OCM selection. **a** The relationship between family members; (**b**) The relationship with neighbours; (**c**) The main medical insurance they have. MOA: Middle-aged and older adults; HOCM: Home-based old-age care mode; COCM: Community-family old-age care mode; Urban employee basic medical insurance: In China, it refers to basic medical insurance program mandated by law, in which all urban employees must enroll. The insurance premium shall be borne by both the employer and the employee; Basic medical insurance for residents: It is a kind of basic medical insurance for residents. Insurance premiums are mainly paid by individual residents (families), supplemented by appropriate government subsidies

The MOA had the highest evaluation of home-based OCM, but the evaluation of home-based OCM was only 3.61, 3.31, 3.48, 2.90 for those with generally and harmonious relationship with their families, or with harmonious and very harmonious relationship with their neighbors. Those whose main insurance type was resident insurance rated the four types of OCM as 3.91, 3.67, 3.62, and 3.53, respectively see Fig. 1.

### Analysis on the influencing factors of the choice of OMC for MOA

#### Basic characteristics and external support received factors

The LR method was used to analyze the influencing factors of MOA’ basic characteristics and external support received for OCM selection. According to the results of univariate analysis and existing old-age care services [17–19], age, residence, educational level, marital status, affordable medical and pension costs, number of children, relationship between family members, and relationship with neighbors were included in the regression model as independent variables. This study divides the evaluation of MOA on the choice of OCM into two categories, and the results of each indicator were categorized and coded into 0 or 1 according to the score. See Table 4 for values assigned to variables.

**Table 4:** Variables assignment

Variables	Assignment
Old-age care mode	Very appropriate/Appropriate = 0, Generally/Inappropriate/Very inappropriate = 1
Age	40 < Age < = 55 = 1, 55 < Age < = 70 = 2, Age > 70 = 3
Residence	Urban = 0, Rural = 1
Education level	Illiteracy = 1, Primary school = 2, Junior high school = 3, High school/Technical school = 4, College degree and above = 5
Marital status	Married = 1, Unmarried or divorced, et al. = 2, Widowed = 3,
Affordable medical and pension costs	500and below = 1, 501–1000 = 2, 1001–1500 = 3, 1501and above = 4
Number of children	No = 1, One = 2, Two = 3, Three = 4, Fore and above = 5
Relationship between family members	Very discordant = 1, Discordant = 2, Generally = 3, Harmonious = 4, Very harmonious = 5
Relationship with neighbors	Very discordant = 1, Discordant = 2, Generally = 3, Harmonious = 4, Very harmonious = 5

The results of regression analysis showed that the choice of OCM for MOA was mainly affected by the education level, number of children, relationship between family members, and neighborhood relationships (*P* < 0.05). In terms of home-based OCM, the degree of inappropriateness among those with a highly discordant relationship with their neighbors was 9.98 times that of those with a very harmonious relationship with their neighbors (*OR* = 9.89, *95%CI* = 2.274 ~ 43.014). The probability of not choosing community-family OCM was 2.506, 2.255, and 1.964 times greater for illiterate, elementary school, and junior high school seniors, respectively, than for seniors with a college and higher education level (*OR* = 2.506, *95%CI* = 1.317 ~ 4.768; *OR* = 2.255, *95%CI* = 1.263 ~ 4.026; *OR* = 1.964, *95%CI* = 1.103 ~ 3.497). Age, marriage status, and affordable medical and pension costs had no statistically significant effect on the choice of OCM (*P* > 0.05) see Table 5.

**Table 5:** Logistic Regression (LR) results

Indicators	HOCM^a^		COCM^b^		Retirement village		Nursing homes	
	*β*	*OR (95%CI)*	*β*	*OR (95%CI)*	*β*	*OR (95%CI)*	*β*	*OR (95%CI)*
**Age (> 70 years)**	-	-	-	-	-	-	-	-
40 < Age < = 55	0.279	1.322(0.744 ~ 2.346)	0.175	1.191(0.757 ~ 1.876)	0.316	1.372(0.872 ~ 2.159)	0.179	1.196(0.767 ~ 1.863)
55 < Age < = 70	0.208	1.231(0.727 ~ 2.084)	-0.171	0.842(0.561 ~ 1.266)	-0.066	0.936(0.622 ~ 1.410)	0.026	1.027(0.689 ~ 1.529)
**Residence (Rural)**	-0.181	0.834(0.578 ~ 1.205)	-0.396***	0.673(0.502 ~ 0.902)	0.087	1.091(0.814 ~ 1.462)	0.055	1.056(0.794 ~ 1.405)
**Education level (College degree and above)**	-	-	-**^c^	-	-***	-	-	-
Illiteracy	-0.155	0.857(0.400 ~ 1.832)	0.919***	2.506(1.317 ~ 4.768)	0.645	1.906(0.999 ~ 3.640)	0.314	1.369(0.752 ~ 2.493)
Primary school	-0.159	0.853(0.436 ~ 1.671)	0.813***	2.255(1.263 ~ 4.026)	0.681**	1.976(1.102 ~ 3.544)	0.523	1.687(0.990 ~ 2.873)
Junior high school	-0.069	0.934(0.483 ~ 1.805)	0.675**	1.964(1.103 ~ 3.497)	0.790***	2.204(1.235 ~ 3.934)	0.475	1.609(0.946 ~ 2.737)
High school/Technical school	-0.136	0.873(0.425 ~ 1.794)	0.204	1.226(0.647 ~ 2.321)	0.024	1.024(0.531 ~ 1.974)	0.102	1.108(0.619 ~ 1.983)
**Marital status (Widowed)**	-	-	-	-	-	-	-	-
Married	-0.101	0.904(0.523 ~ 1.560)	-0.338	0.713(0.467 ~ 1.089)	-0.320	0.726(0.476 ~ 1.109)	-0.352	0.703(0.458 ~ 1.081)
Unmarried or divorced, et al	0.024	1.025(0.458 ~ 2.294)	-0.198	0.820(0.405 ~ 1.663)	0.008	1.008(0.496 ~ 2.047)	-0.213	0.809(0.400 ~ 1.636)
**Affordable medical and pension costs (1501and above)**	-	-	-	-	-	-	-	-
500 and below	-0.162	0.850(0.485 ~ 1.491)	0.039	1.040(0.673 ~ 1.607)	0.495**	1.640(1.061 ~ 2.535)	0.321	1.378(0.902 ~ 2.105)
501–1000	-0.112	0.894(0.548 ~ 1.458)	0.284	1.329(0.902 ~ 1.957)	0.385	1.469(0.989 ~ 2.183)	0.342	1.408(0.964 ~ 2.056)
1001–1500	0.087	1.091(0.656 ~ 1.816)	-0.079	0.924(0.605 ~ 1.412)	0.193	1.212(0.788 ~ 1.864)	0.094	1.099(0.731 ~ 1.651)
**Number of children (Fore and above)**	-	-	-	-	-	-	-	-
No	1.335**	3.801(1.206 ~ 11.972)	-0.065	0.937(0.370 ~ 2.370)	-0.622	0.537(0.204 ~ 1.417)	-0.374	0.688(0.279 ~ 1.694)
One	0.824	2.280(0.976 ~ 5.328)	-0.231	0.794(0.444 ~ 1.419)	-0.327	0.721(0.404 ~ 1.288)	-0.768***	0.464(0.260 ~ 0.827)
Two	0.962**	2.618(1.225 ~ 5.595)	0.043	1.044(0.643 ~ 1.695)	-0.194	0.823(0.509 ~ 1.332)	-0.548**	0.578(0.353 ~ 0.945)
Three and above	0.808**	2.242(1.040 ~ 4.836)	-0.202	0.817(0.500 ~ 1.334)	-0.295	0.745(0.458 ~ 1.212)	-0.567**	0.567(0.345 ~ 0.934)
**Relationship between family members (Very harmonious)**	-***	-	-	-	-	-	-	-
Very discordant	0.936	2.550(0.663 ~ 9.813)	0.694	2.001(0.557 ~ 7.192)	-0.373	0.689(0.183 ~ 2.594)	-0.529	0.589(0.165 ~ 2.108)
Discordant	1.436***	4.204(1.641 ~ 10.769)	-0.068	0.935(0.388 ~ 2.249)	-0.165	0.848(0.345 ~ 2.088)	-0.410	0.664(0.274 ~ 1.608)
Generally	1.287***	3.623(1.887 ~ 6.955)	0.116	1.123(0.628 ~ 2.006)	-0.270	0.763(0.421 ~ 1.384)	-0.698**	0.498(0.278 ~ 0.891)
Harmonious	0.291	1.337(0.825 ~ 2.167)	0.377**	1.458(1.007 ~ 2.111)	0.242	1.274(0.878 ~ 1.849)	-0.181	0.835(0.578 ~ 1.204)
**Relationship with neighbors (Very harmonious)**	-***	-	-**	-	-	-	-***	-
Very discordant	2.292***	9.89(2.274 ~ 43.014)	0.991	2.695(0.673 ~ 10.792)	0.805	2.237(0.566 ~ 8.840)	1.133	3.105(0.769 ~ 12.541)
Discordant	1.238**	3.449(1.239 ~ 9.596)	0.873	2.394(0.928 ~ 6.170)	0.050	1.051(0.381 ~ 2.904)	1.075**	2.929(1.133 ~ 7.569)
Generally	0.389	1.476(0.785 ~ 2.773)	0.845***	2.327(1.425 ~ 3.800)	0.55**	1.734(1.061 ~ 2.833)	1.388***	4.006(2.436 ~ 6.586)
Harmonious	0.476	1.610(0.959 ~ 2.702)	0.468**	1.597(1.079 ~ 2.363)	0.284	1.328(0.897 ~ 1.967)	0.826***	2.284(1.563 ~ 3.338)
**Constant**	-2.661***	0.070	-0.765	0.465	-1.437***	0.238	-0.347	0.707

^a^*HOCM* Home-based old-age care mode

^b^*COCM* Community-family old-age care mode

^c^***, **, * indicate significant at 0.01, 0.05 and 0.1 levels, respectively

#### Concentration analysis of the choice of OCM for MOA based on personal health status and POA

The physical health status, self-care ability, disease status, and personal POA of MOA were ranked from low to high, while the individuals who will mainly care for them when they got sick were categorized as “themselves”, “spouses”, “children”, “relatives or others”. The analyses indicate that the evaluations of four types of OCM were basically equal among people with different conditions (Concentration index = 0.0895 ~ 0.0166). And the evaluation of the choice of four types of OCM based on personal POA had the lowest concentration (Concentration index = 0.0606, 0.0647, 0.0715, 0.0895) (Table 6).

**Table 6:** Concentration index analysis of old-age care mode selection evaluation based on health status and perspectives on old-age

Indicators	Old-age care modes (Concentration index)			
	HOAC^a^	PAHC^b^	Retirement village	Nursing homes
Physical health	0.0248	0.0343	0.0255	0.0340
Self-care skills	0.0168	0.0199	0.0077	0.0166
Disease	0.0285	0.0179	0.0273	0.0211
Caregiver^c^	0.0207	0.0309	0.0177	0.0258
Perspectives on old-age	0.0606	0.0647	0.0715	0.0895

^a^*HOAC* Home-based older adult care

^b^*PAHC* Providing for the aged at home by communities

^c^Caregiver: Who will take care for them when they got sick

### Effect manner and scope of influencing factors of the choice of OCM

The current study also analyzed the mediating effect of personal POA on the effect of personal characteristics and external support factors on the choice of OCM. The mediating effect of MOA’ POA ranges from -0.002 to 0.013, the mediating effect of choosing home-based OCM as relatively the largest, and the mediating effect of choosing retirement village OCM was relatively the smallest. Specific to each influencing factor, the mediating effect of POA on the relationship with neighbors affecting the choice of OCM was greater than 0.007, and it has a masking effect on the relationship between the number of children and the choice of OCM (Indirect effect value < 0) see Table 7.

**Table 7:** The mediating effect of personal perspectives on the effect of health status and external support on the evaluation of OCM^a^ selection

Affect way^d^		*β*	*SE*	*t*	*P*	*(LLCI, ULCI)*	Total effect	Indirect effect	*BootSE*	*(BootLLCI, BootULCI)*	Effect category
M → × 1 → Y1	× 1 → M^b^	0.014	0.008	1.837	0.067	(-0.001, 0.029)	0.067***	-	-	(-0.001, 0.001)	Indirect effect is not significant
	× 1 → Y1	0.001	0.023	0.056	0.955	(-0.044, 0.047)					
	M → Y1^c^	0.067	0.005	12.654	< 0.001	(0.057, 0.078)					
M → × 2 → Y1	× 2 → M^b^	0.012	0.006	1.856	0.064	(-0.001, 0.025)		-	-	(-0.001, 0.001)	Indirect effect is not significant
	× 2 → Y1	0.007	0.027	0.269	0.788	(-0.046, 0.061)					
	M → Y1	0.067	0.005	12.641	< 0.001	(0.057, 0.078)					
M → × 3 → Y1	× 3 → M^b^	0.053	0.006	9.477	< 0.001	(0.042, 0.064)		0.012	0.003	(0.007, 0.017)	Mediation effect
	× 3 → Y1	0.226	0.031	7.335	< 0.001	(0.166, 0.287)					
	M → Y1	0.055	0.005	10.247	< 0.001	(0.045, 0.066)					
M → × 4 → Y1	× 4 → M^b^	0.069	0.005	12.807	< 0.001	(0.058, 0.080)		0.013	0.003	(0.007, 0.018)	Mediation effect
	× 4 → Y1	0.178	0.032	5.543	< 0.001	(0.115, 0.241)					
	M → Y1	0.054	0.006	9.695	< 0.001	(0.044, 0.066)					
M → × 1 → Y2	× 1 → Y2	0.098	0.025	3.943	< 0.001	(0.049, 0.147)	0.070***	0.001	0.001	(0.001, 0.003)	Mediation effect
	M → Y2	0.069	0.006	12.022	< 0.001	(0.058, 0.080)					
M → × 2 → Y2	× 2 → Y2	-0.109	0.029	-3.707	< 0.001	(-0.166, -0.051)		-0.001	0.001	(-0.003, -0.001)	Masking effect
	M → Y2	0.071	0.006	12.478	< 0.001	(0.060, 0.083)					
M → × 3 → Y2	× 3 → Y2	0.064	0.034	1.863	0.063	(-0.003, 0.132)		0.003	0.002	(-0.001, 0.008)	Mediation effect
	M → Y2	0.067	0.006	11.081	< 0.001	(0.055, 0.079)					
M → × 4 → Y2	× 4 → Y2	0.145	0.035	4.100	< 0.001	(0.075, 0.214)		0.010	0.003	(0.004, 0.016)	Mediation effect
	M → Y2	0.060	0.006	9.707	< 0.001	(0.048, 0.073)					
M → × 1 → Y3	× 1 → Y3	0.073	0.027	2.705	0.007	(0.020, 0.126)	0.044***	0.001	0.001	(0.001, 0.003)	Mediation effect
	M → Y3	0.043	0.006	6.854	< 0.001	(0.030, 0.055)					
M → × 2 → Y3	× 2 → Y3	-0.136	0.032	-4.317	< 0.001	(-0.198, -0.074)		-0.002	0.001	(-0.004, -0.001)	Masking effect
	M → Y3	0.046	0.006	7.328	< 0.001	(0.033, 0.057)					
M → × 3 → Y3	× 3 → Y3	0.039	0.037	1.049	0.295	(-0.034, 0.112)		0.002	0.002	(-0.003, 0.006)	Mediation effect Mediation effect
	M → Y3	0.042	0.007	6.370	< 0.001	(0.029, 0.054)					
M → × 4 → Y3	X4 → Y4	0.100	0.038	2.608	0.009	(0.025, 0.175)		0.007	0.003	(0.001, 0.013)	Mediation effect Mediation effect
	M → Y3	0.037	0.007	5.448	< 0.001	(0.023, 0.050)					
M → × 1 → Y4	× 1 → Y4	0.076	0.029	2.688	0.008	(0.020, 0.132)	0.058***	0.001	0.001	(0.001, 0.003)	Mediation effect
	M → Y4	0.057	0.007	8.712	< 0.001	(0.044, 0.070)					
M → × 2 → Y4	× 2 → Y4	-0.134	0.033	-4.016	< 0.001	(-0.200, 0.069)		-0.002	0.001	(-0.004, -0.001)	Masking effect
	M → Y4	0.060	0.007	9.165	< 0.001	(0.047, 0.073)					
M → × 3 → Y4	× 3 → Y4	0.023	0.039	0.590	0.556	(-0.054, 0.100)		0.001	0.002	(-0.003, 0.005)	Mediation effect Mediation effect
	M → Y4	0.057	0.007	8.272	< 0.001	(0.044, 0.071)					
M → × 4 → Y4	× 4 → Y4	0.148	0.040	3.682	< 0.001	(0.069, 0.227)		0.010	0.003	(0.004, 0.016)	Mediation effect
	M → Y4	0.048	0.007	6.769	< 0.001	(0.034, 0.062)					

^a^*OCM* Old-age care mode

^b^x1 refers to the education level, × 2 refers to the number of children, × 3 refers to the relationship between family members, × 4 refers to the relationship with neighbors, Y1 refers to Home-based older adults care, Y2 refers to refers to Providing for the aged at home by communities, Y3 refers to the selection of Retirement village, Y4 refers to the selection of Nursing homes, and M refers to the individual’s perspectives on old-age; ^b^: For different old-aged modes, the results of M → × 1, M → × 2, M → × 3, M → × 4 are all equal. In order to reduce the size of the table, only the values of these four relationships are listed in home-based older adult care

^c^Indicates the direct effect

^d^The control variables in the analysis are age, marriage, and affordable expenses

## Discussion

The purpose of this study was to evaluate the status and influencing factors of the choice of OCM through the analysis of the survey data on the willingness to support older adults in Henan Province, China, and to explore the manner and scope of the influencing factors. The survey results showed that the overall score of MOA on the choice of OCM was higher than 3.49 (Full score = 5), which was mainly related to the education level, number of children, relationship between family members and relationship with neighbors. The allocation of OCM selection based on personal POA was relatively the largest, and POA played a mediating effect on the influence factors such as educational level on the choice of OCM for MOA (Mediating effect was -0.002 ~ 0.013).

In terms of OCM preferences, the survey found that middle-aged and older adults (MOA) displayed a higher inclination towards choosing home-based OCM compared to nursing home OCM. Interestingly, there was minimal disparity in the willingness of middle-aged individuals compared to older adults when it came to selecting nursing home OCM. The findings align with the research conducted by Fan LQ et al. [36], which suggests that home-based OCM is preferred by the majority of middle-aged and older adults (MOA) due to emotional benefits, the familiarity of the living environment, and personalized care. On the other hand, nursing homes OCM offers advantages in terms of medical service provision [36]. The evaluations provided by middle-aged and older adults (MOA) regarding the selection of community-family and retirement village OCM were rated at 3.70 and 3.72, respectively. Notably, these evaluations displayed a pattern that decreased initially with the age of older adults and then increased [11]. These findings collectively indicate that the changing dynamics of the economy and society have presented challenges to the traditional model of home-based OCM. Factors such as smaller family sizes, longer distances between family members, and younger individuals working longer hours have contributed to a situation where more older adults are compelled to explore non-home-based care options. The evolving socio-economic landscape has necessitated a shift in the way care is provided to older adults who may not have the option of relying solely on home-based care [37]. In addition, as a novel type of OCM, the evaluation of community-family OCM may have been influenced by the brief period of development in China and the non-standard structure, administration, operation mechanisms and methods of relevant supply institutions.

The findings also revealed that the MOA’ evaluation of the OCM was dropping as their relationships with their neighbors deteriorated. For example, the results of this study showed that people who had a very discordant relationship with their neighbors were 9.98 times more likely to think that home-based OCM was inappropriate than those who had a very harmonious relationship with their neighbors. The same trend was shown in the relationships between family members. On the other hand, the evaluation of MOA on OCM increases with the improvement of their education level, and with the continuous improvement of education level, this change showed a rapid upward trend, which is most obvious in community-family OCM. This may be related to the fact that students in junior high school and below mainly learn the basic knowledge of life and society, while the learning in high school/technical school and above greatly improves students’ ability to acquire knowledge [38]. The current study also found that, compared with families with many children, older adults with fewer children bear greater old-age care risks.

In addition, this study indicated that there were substantial disparities in the POA of MOA for individuals who select different OCMs, but there were no significant differences in their attitudes regarding pricing and spending, regardless of whether they select retirement villages or nursing homes OCMs (*P* > 0.05). A possible explanation is that the cost of the people who choose the retirement village and nursing homes is mainly borne by their children, and older adults do not know the price and cost [39]. In addition, the score for the importance of basic diet and daily life was relatively high, and the score of other people’s opinion was relatively low. This may be due to the fact that aging persons are increasingly worried about the quality of their everyday life when their own health status declines and their social engagement declines [16]. This suggests that policy makers and old-age care institutions could prioritize changing the POA of MOA through publicity and education, and provide them with high-quality and comprehensive daily services to ensure their quality of life in old-age.

Finally, the mediating effect of the personal POA of MOA on the influence of personal characteristics and external support factors on the evaluation of OCM selection was between -0.002 and 0.013, which reminds that the choice of OCM is related to multiple factors at the same time, and no single feature alone can determine a person’s final choice [40]. Moreover, the personal POA has a masking effect on the relationship between the number of children and the choice of OCM. The current study recommends that when developing policies and delivering services, both the government and old-age care institutions should prioritize the diverse perspectives and varying health and social statuses of middle-aged and older adults (MOA). Instead of relying exclusively on metrics such as the number of children and economic status, it is essential to take a more comprehensive approach.

This study has several advantages: Firstly, the authors focus the study direction on the choice of OCM for MOA, and includes and compares the four existing OCMs. Secondly, the study comprehensively explored the influence of personal basic characteristics, health status, POA and external support received in their choices. Thirdly, based on the mediating impact, this study investigated the manner and extent of influencing factors on the decision to select a particular type of OCM. However, a significant limitation of this study is that the results of the study based on the age of the mean segment setting fail to fully capture the differences in OCM choice between the middle-aged and the older adults. In addition, methods such as the Concentration Index and Mediation Effect model used in this study have high requirements for the quantity and quality of data, limiting their applicability to other similar investigations. Thirdly, given the cross-sectional nature of this study, the authors believe that there may be other important confounding factors, such as OCM used by an older adult and long-term socioeconomic development and ideological changes, which deserve further exploration and analysis.

## Conclusions

This study evaluated the current status and influencing factors of OCM choice among MOA in Henan Province, China, and explored the effect manner and scope of the influencing factors. The results showed that the evaluation of choosing a non-home OCM was generally, and the choice of OCM was mainly related to the education level, number of children, relationship between family members, relationship with neighbors, and individual’s POA. At the same time, POA played a mediating role in the influence of factors such as education level on the choice of OCM for MOA. Therefore, this study suggest that policymakers could promote the choice of non-home OCM by taking measures such as improving the relationship between MOA and changing their POA. On the other hand, when the government formulates relevant policies and provides old-age services, it could pay more attention to the perspectives of MOA, and provide them with high-quality and comprehensive daily services to ensure their quality of life in the old-age.

### Supplementary Information

**Additional file 1.**

## Data Availability

The datasets used and analyzed during the current study are available from the corresponding author on reasonable request.

## Abbreviations

OCM: Old-age care mode
MOA: Middle-aged and older adults
POA: Perspectives on old-age
LR: Logistic regression
