# Supplementary material for: Willingness and influencing factors of old-age care mode selection among middle-aged and older adults in Henan Province, China
Source: BMC Geriatr. 2024 Jan 18;24:72. doi: 10.1186/s12877-023-04559-w (PMC10797948; doi:10.1186/s12877-023-04559-w)
Supplement: Supplementary file 1 — Additional file 1. [file 12877_2023_4559_MOESM1_ESM.doc]

**Survey on the willingness of old-age care for the middle-aged and older adults in Henan Province**

We are conducting a survey on the willingness and influencing factors of old-age care mode selection, and we would like to ask for your support. There is no right or wrong answer, just a true reflection of your wishes, and your answers will be kept strictly confidential. Thank you for your co-operation and support.

**1. Personal characteristics**

**A1** Primary place of residence: ① Urban ② Rural

**A2** Your gender: ① Male ② Female

**A3** Your age: ( ) years.

**A4** Education level:

① No schooling ② Primary school

③ Junior high school ④ High school/Technical school

⑤ College degree and above

**A5** Marital status:

① Married ② Divorced ③ Widowed ④ Unmarried ⑤ Others

**A6** How many children do you have? (Living children, including adopted, fostered):

① No ② One ③ Two ④ Three ⑤ Fore and above

**A7** How much you can afford to spend on medical and pension costs each month?

① 500 and below ② 501-1000 ③ 1001-1500

④ 1501-2000 ⑤ 2001-2500 ⑥ 2501 and above

**2. Health status and external support**

**B1** Physical health status:

① Very good ② Good ③ Generally ④ Poor ⑤ Very poor

**B2** Can you look after yourself? ( )

① Fully self-care ② Some rely on others ③ Dependent on others

**B3** Your illness status? ( )

① Not sick ② One ③ Two ④ Three ⑤ Four and more

**B4** If ill, who is the primary caregiver?

① Own ② Spouse ③ Children ④ Relatives or others

**B5** What are the relationships between family members?

① Very discordant ② Discordant ③ Generally

④ Harmonious ⑤ Very harmonious

**B6** What is your relationship with your neighbors around you?

① Very discordant ② Discordant ③ Generally

④ Harmonious ⑤ Very harmonious

**B7** Types of major medical insurance coverage available:

① Urban employee basic medical insurance

② Basic medical insurance for residents

③ Commercial insurance

④ others

**3. Perspectives on old-age**

**C1** The importance of basic diet and daily life:

① Very important ② important ③ Generally ④ unimportant ⑤ No impact

**C2** The importance of cultural and recreational activities:

① Very important ② important ③ Generally ④ unimportant ⑤ No impact

**C3** The importance of professional medical care:

① Very important ② important ③ Generally ④ unimportant ⑤ No impact

**C4** The importance of “raising children to prevent aging”:

① Very important ② important ③ Generally ④ unimportant ⑤ No impact

**C5** The importance of service attitude and quality:

① Very important ② important ③ Generally ④ unimportant ⑤ No impact

**C6** The importance of price and expenditure:

① Very important ② important ③ Generally ④ unimportant ⑤ No impact

**C7** The importance of national pension policy:

① Very important ② important ③ Generally ④ unimportant ⑤ No impact

**C8** The importance of family ideas:

① Very important ② important ③ Generally ④ unimportant ⑤ No impact

**C9** The importance of other people's opinion:

① Very important ② important ③ Generally ④ unimportant ⑤ No impact

**4. Evaluation of old-age care mode choices**

**D1** To what extent do you think it is appropriate to choose home-based approach (Living at home, with family members or others caring for the older adult in the home)to ageing ?

① Very appropriate ② Appropriate ③ Generally

④ Inappropriate ⑤ Very inappropriate

**D2** To what extent do you think it is appropriate to choose community-family approach to ageing?

① Very appropriate ② Appropriate ③ Generally

④ Inappropriate ⑤ Very inappropriate

**D3** To what extent do you think it is appropriate to choose retirement village approach to ageing?

① Very appropriate ② Appropriate ③ Generally

④ Inappropriate ⑤ Very inappropriate

**D4** To what extent do you think it is appropriate to choose a nursing homes approach to ageing?

① Very appropriate ② Appropriate ③ Generally

④ Inappropriate ⑤ Very inappropriate
